# *Malassezia*‐Induced Type 2 Immunity in Head Neck Shoulder Type Atopic Dermatitis

**DOI:** 10.1111/all.70302

**Published:** 2026-03-24

**Authors:** I. Suhrkamp, T. Heidland, C. Saft, A. K. Kamps, S. Edenhart, O. Kniemeyer, W. Francuzik, S. Weidinger, P. Bacher, C. Bang, G. Heine

**Affiliations:** ^1^ Department of Dermatology, Venereology and Allergy University Hospital Schleswig‐Holstein Kiel Germany; ^2^ Institute of Immunology University Hospital Schleswig‐Holstein Kiel Germany; ^3^ Department of Molecular and Applied Microbiology Leibniz Institute for Natural Product Research and Infection Biology (Leibniz‐HKI) Jena Germany; ^4^ Department of Dermatology, Venereology and Allergy Charité – Universitätsmedizin Berlin Berlin Germany; ^5^ Institute of Clinical Molecular Biology Christian‐Albrechts‐University Kiel Kiel Germany

**Keywords:** atopic dermatitis, head neck shoulder, Malassezia spp

To the Editor,

Atopic dermatitis (AD) is an inflammatory skin disease characterized by a skin barrier defect, type II sensitization and microbial dysbiosis [1]. *Malassezia* spp. colonize mainly the scalp, face and upper trunk [2]. In nonatopic individuals (NA), the immune response to *Malassezia* spp. is of a Th1/Th17 type and induces IgG/IgA [3]. Patients with AD with head and neck dermatitis (HND) often show *Malassezia*‐specific IgE antibodies [2]. Only a few data are available on the interplay of AD skin and type II immunity against *Malassezia* spp., *including Malassezia furfur* (*M. furfur*), which was chosen here as a representative species based on previous research [2].

We investigated the allergen‐specific cellular immune response regarding basophil activation, the T‐cell phenotype and the immunoglobulin response in HND donors with *M. furfur*‐specific‐IgE (*M. furfur*‐IgE) in comparison to NA. The basophil activation test (BAT), a sensitive functional test for IgE sensitization, was positive in HND but not in NA, with a median 800‐fold lower effective concentration (EC) 50 (Figure 1A,B, Figure S1A). The response against *M. furfur* cell extracts was stronger than against *M. furfur*‐secreted proteins (Figure S1B). The *M. furfur*‐BAT EC50 values negatively correlate with serum *M. furfur*‐IgE in the HND group (Figure S1C), accordingly, AD patients with *M. furfur*‐IgE below detection thresholds were *M. furfur*‐BAT negative (Figure S1D).

**FIGURE 1:**
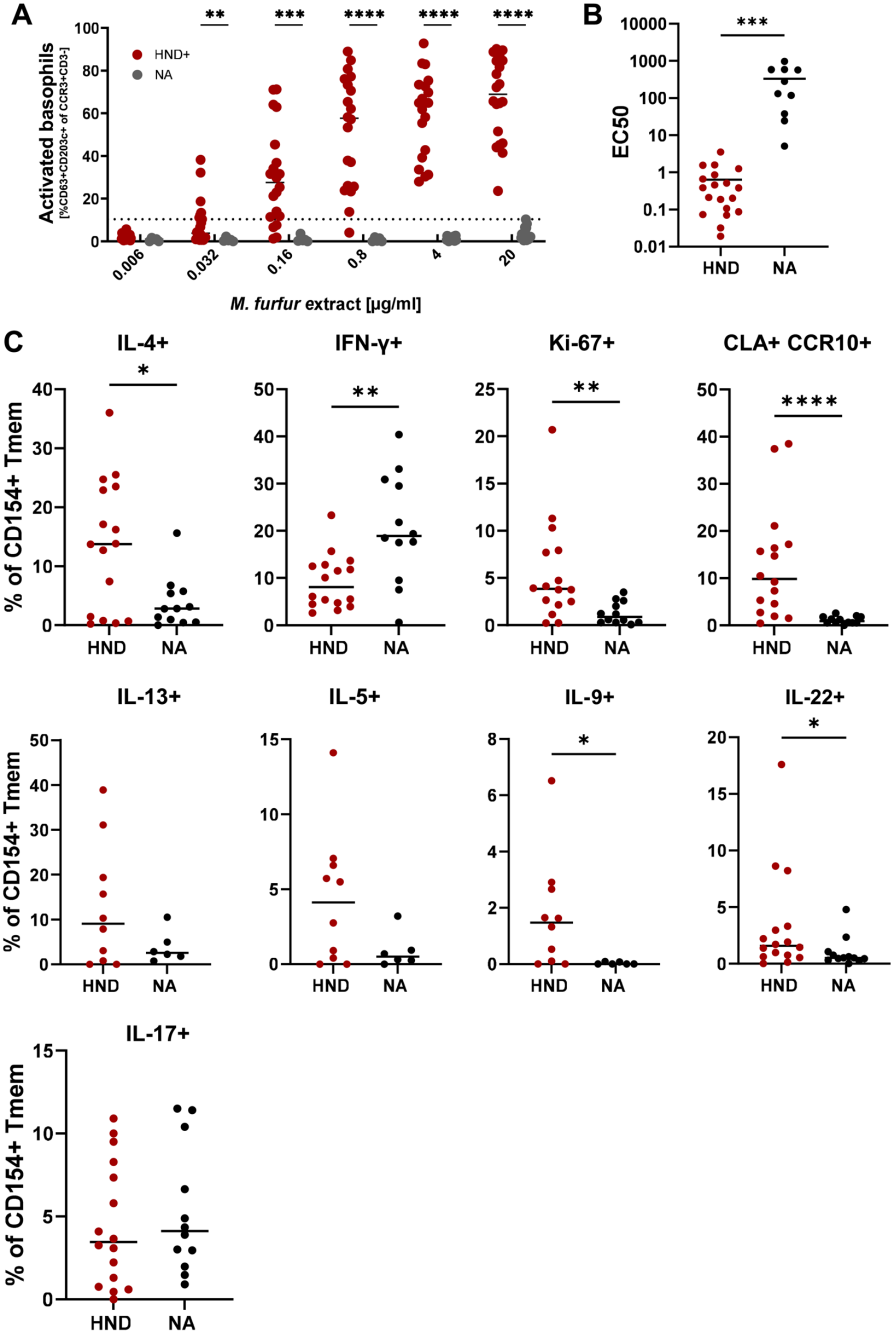
*M. furfur*‐specific cellular response in HND. Peripheral blood cells from HND and healthy controls (NA) were analyzed by flow cytometry. (A) Frequency of activated basophils in response to titrated *M. furfur*‐extract (*n* = 20). (B) *Malassezia* half‐maximal concentration threshold (EC50) activating basophils. *n* = 19 HND, 10 NA, ****p < 0.0001 (unpaired *t*‐test). (C) Antigen‐reactive T‐cell enrichment (ARTE) following *M. furfur*‐specific stimulation analyzed regarding expression of cytokines, proliferation (Ki‐67 antigen), homing markers (CLA, CCR10). Dot plots represent individual donors with median, *n* = 6–16, *p < 0.05; **p < 0.01; ***p < 0.001 (Mann–Whitney *U* test).

Specific IgE largely depends on IL‐4 or IL‐13 from Th2 cells [4]. In HND, *M. furfur*‐specific T helper memory (CD4+ CD45RA‐ CD45RO+, Tmem) cells were detected by CD154 upregulation (*M. furfur* Tmem) using the ARTE method [5]. These cells predominantly expressed typical Th2 cytokines including IL‐4, IL‐13, IL‐5, and IL‐9 but also IL‐22 and IFN‐γ, the last was increased in T cells from NA (Figure 1C). However, an IL‐17 response to *M. furfur* by NA [3] was not confirmed in our data. Recent proliferation and skin homing was suggested in *M. furfur*‐Tmem from HND and not NA donors by higher frequencies of Ki‐67+ and CLA/CCR10+ cells (Figure 1C, Figure S2E). Thus, in HND a type II inflammation is suggested by expression of functional *M. furfur*‐IgE and the presence of proliferating, cytokine‐producing, skin‐homing Th2 cells (Figure S2D). The HND severity determined by SCORAD was not correlated with basophil EC50 values or CLA/CCR10+ T cells suggesting that our findings rather reflect an immunologic endotype than an inflammation‐induced short‐lived phenomenon.

To determine whether an immunodominant allergen from *M. furfur* is prevalent in HND, we performed ELISA and western blot (WB) analysis with patients' sera against *M. furfur*. *M. furfur*‐IgE was restricted to the HND group and not detectable in NA by ELISA (Figure 2A). In HND, the numbers of different *M. furfur*‐specific proteins in WB analysis were independent from the serum‐specific IgE concentrations (Figure 2B, Figure C). In contrast, the overall *M. furfur*‐specific IgA and ‐IgG serum levels were comparable between both groups (Figure 2A). Whether the abundance of *M. furfur* on the skin impacts the sensitivity to *M. furfur* proteins was investigated by qPCR. Data confirm an increased *Malassezia* abundance in the sebaceous interscapular region compared with the cubital fossa in all individuals, which was comparable between HND and NA groups (Figure 2D, Figure E). The predominant abundances observed were *M. restricta*, *M. sympodialis*, and *M. globosa* with *M. furfur* below detection threshold in most HND and NA (Figure 2E), in line with previously published work [6]. The strong IgE‐reactivity against *M. furfur* extract may be explained by its high cross‐reactivity with *Malassezia* spp., which was confirmed by ELISA (Figure S3).

**FIGURE 2:**
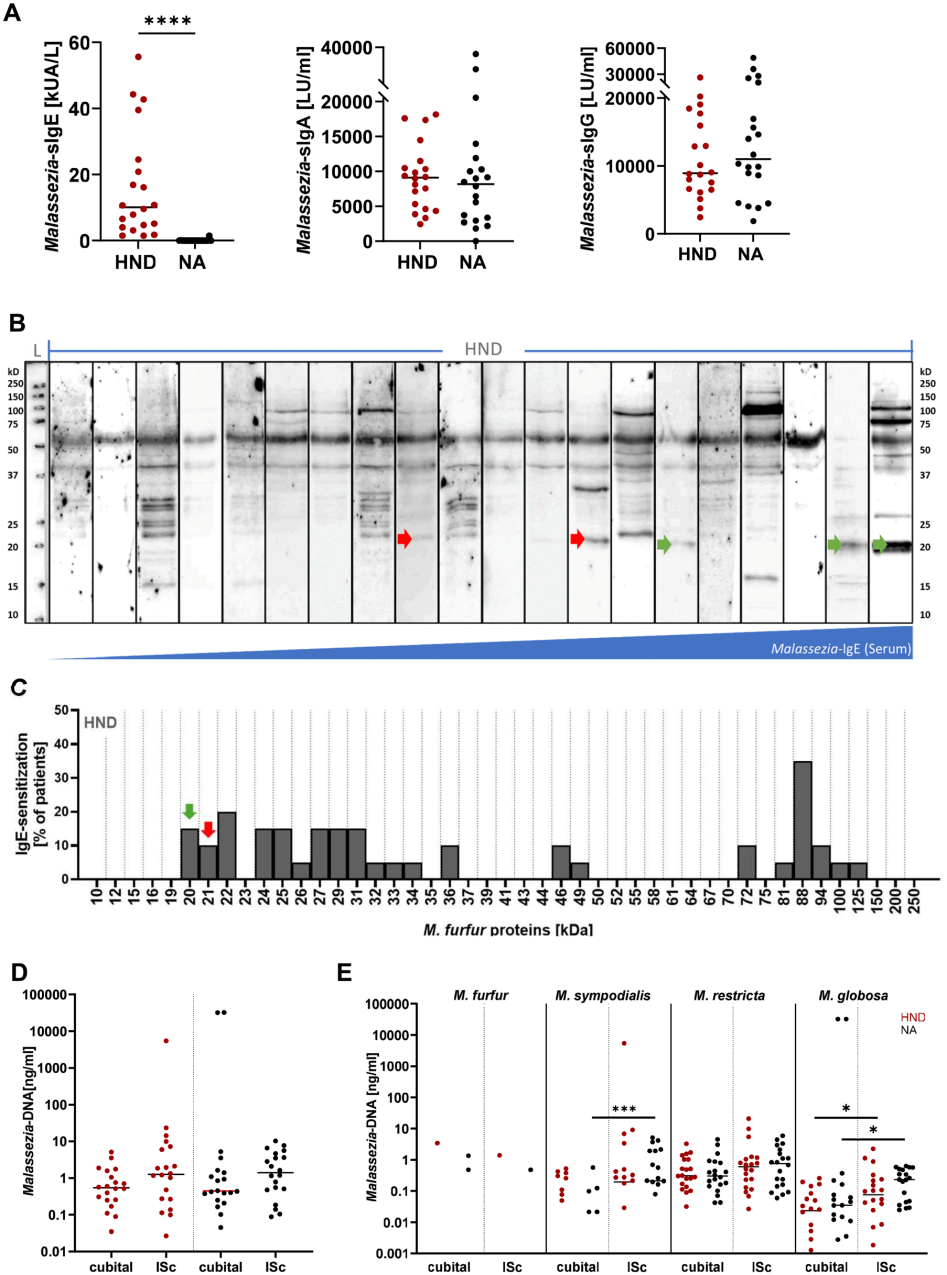
*Malassezia*‐specific IgE response and skin colonization. (A) Concentration of *M. furfur*‐specific IgE, IgA, and IgG in serum of HND patients and NA (*n* = 20), ****p < 0,0001. (B) IgE WB of *M. furfur* extract with HND patient sera displayed according to the specific serum IgE concentrations from low to high. Each column represents one HND patient (*n* = 20). (C) Statistical analysis of sensitization against *M. furfur‐*specific proteins. % HND patients showing a band at the respective kDA size (*n* = 20). Green = Mala f2 (20 kDa), red = Mala f3 (21kDa). *Malassezia*‐load in HND (red) and NA (black) on cubital fossa and intrascapular (ISc) skin with (D) total *Malassezia* spp. and (E) subspecies‐specific DNA load. (*n* = 20), *p < 0.05; ***p < 0.001 (Mann–Whitney *U* test).

Our data show a functional specific IgE and specific Th2 response toward *Malassezia* spp. in HND and not NA. The contact of *Malassezia* proteins with specific IgE‐loaded effector cells and specific Th2 cells in the skin may initiate and maintain skin damage resulting in HND as phenotype caused by type II‐inflammation against a commensal [7]. Persistent depletion of *Malassezia* spp., for example, by prolonged antifungal treatment may avoid the need for systemic treatment in mild AD or increase the treatment efficiency in severe AD by reducing the local *Malassezia‐*specific type II response.

## Author Contributions

Conception, design, and funding: G.H., C.B., P.B. Performed experiments and produced reagents: T.H., C.S., A.K.K., I.S., S.E., O.K. Analysis and/or interpretation: I.S., F.W., S.W., P.B., C.B., G.H. All authors wrote and gave consent to the manuscript.

## Funding

Funding sources: none. G.H. receives funding from the Deutsche Forschungsgemeinschaft (DFG, grant #454193335—SFB 1526‐A02 and ‐S01)*—Project‐ID 454193335—SFB 1526″*. S.E. was supported by the Bundesministerium für Bildung und Forschung (BMBF) InfectControl 2020 Projects AnDiPath 03ZZ0838A. This research as supported by the Deutsche Forschungsgemeinschaft (DFG) under Germany's Excellence Strategy: EXC216‐7 Project ID 390884018 ‘Precision Medicine in Chronic Inflammation’.

## Consent

All authors reviewed the final manuscript version and consented to its submission.

## Conflicts of Interest

The authors declare no conflicts of interest.

## Supporting information


**Figure S1:** *Malassezia*‐induced basophil activation test in HND. Peripheral full blood cells from HND and NA were incubated with titrated *M. furfur* extract and analyzed after erythrolysis by flow cytometry regarding CD63 and CD203 expression on CCR3 + CD3‐ basophils. (A) ROC curve analysis of CD63 + CD203+ cells. (B) Frequency of activated basophils to titrated *M. furfur* extract and *M. furfur* soluble proteins in HND donors. (C) Correlation between EC50 of the basophils activation test and *M. furfur*‐specific serum IgE levels (log_10_) in HND patients. (D) Frequency of activated basophils from sensitized (HND+) and not sensitized (HND‐) individuals with AD in response to titrated *M. furfur*‐extract. Box plots represent the 25–75 percentils with range (5%–95%). *p < 0.05; **p < 0.01; ***p < 0.001; ****p < 0.0001.
**Figure S2:** M. furfur‐reactive memory CD4+ T cells in HND and NA individuals. M. furfur‐Tmem cells were characterized by ARTE. The dot plots are gated on single CD4+ CD45RO+ CD45RA‐ CD154+ lymphocytes. (A) Ex vivo cytokine production. Graphs show one representative HND donor. (B) Absolute and (C) relative frequency of M. furfur‐Tmem. (D) Statistical analysis of cytokine expression by skin homing of M. furfur‐Tmem in HND donors and (E) M. furfur‐Tmem cell frequencies expressing CCR10+ or CLA+ after stimulation compared to baseline (*n* = 10–16) and NA (*n* = 6–12). *p < 0.05; **p < 0.01; ***p < 0.001.
**Figure S3:** Blocking cross‐reactivity between Malassezia spp. Inhibition assay of M. furfur IgE binding by ELISA. Serum was incubated with indicated yeast extracts or serum before added to the ELISA plate. *n* = 10, 7 individuals. Results were normalized to sample with highest inhibition effect. Kruskal–Wallis with * < 0.05; ** < 0.01; *** < 0.001, and **** < 0.0001.

## Data Availability

The data that support the findings of this study are available from the corresponding author upon reasonable request.
