# Supplementary material for: Malassezia‐Induced Type 2 Immunity in Head Neck Shoulder Type Atopic Dermatitis
Source: Allergy. 2026 Mar 24;81(7):2535–8. doi: 10.1111/all.70302 (PMC13342752; doi:10.1111/all.70302)
Supplement: Supplementary file 1 — Figure S1: Malassezia‐induced basophil activation test in HND. Peripheral full blood cells from HND and NA were incubated with titrated M. furfur extract and analyzed after erythrolysis by flow cytometry regarding CD63 and CD203 expression on CCR3 + CD3‐ basophils. (A) ROC curve analysis of CD63 + CD203+ cells. (B) Frequency of activated basophils to titrated M. furfur extract and M. furfur soluble proteins in HND donors. (C) Correlation between EC50 of the basophils activation test and M. furfur‐specific serum IgE levels (log10) in HND patients. (D) Frequency of activated basophils from sensitized (HND+) and not sensitized (HND‐) individuals with AD in response to titrated M. furfur‐extract. Box plots represent the 25–75 percentils with range (5%–95%). *p < 0.05; **p < 0.01; ***p < 0.001; ****p < 0.0001. Figure S2: M. furfur‐reactive memory CD4+ T cells in HND and NA individuals. M. furfur‐Tmem cells were characterized by ARTE. The dot plots are gated on single CD4+ CD45RO+ CD45RA‐ CD154+ lymphocytes. (A) Ex vivo cytokine production. Graphs show one representative HND donor. (B) Absolute and (C) relative frequency of M. furfur‐Tmem. (D) Statistical analysis of cytokine expression by skin homing of M. furfur‐Tmem in HND donors and (E) M. furfur‐Tmem cell frequencies expressing CCR10+ or CLA+ after stimulation compared to baseline (n = 10–16) and NA (n = 6–12). *p < 0.05; **p < 0.01; ***p < 0.001. Figure S3: Blocking cross‐reactivity between Malassezia spp. Inhibition assay of M. furfur IgE binding by ELISA. Serum was incubated with indicated yeast extracts or serum before added to the ELISA plate. n = 10, 7 individuals. Results were normalized to sample with highest inhibition effect. Kruskal–Wallis with * < 0.05; ** < 0.01; *** < 0.001, and **** < 0.0001. [file ALL-81-2535-s001.pdf]

Supplementary information

Materials and Methods

Patients: Atopic dermatitis patients and healthy controls (own or family history for no atopy, NA) were enrolled at the Center for Inflammatory Skin Diseases of the Department of Dermatology at the University Hospital Schleswig-Holstein (UKSH), Campus Kiel. Approval was obtained from the local ethics committee (D519/20). All study participants provided informed written consent. Patients were over 18 years of age and diagnosed with AD. SCORing Atopic Dermatitis Index (SCORAD) was determined at date of inclusion with focus on the head, neck and shoulder area (HND). Healthy controls and patients were matched as closely as possible in terms of age and sex. All participants underwent serological testing for sensitization to *Malassezia furfur* (*M. furfur*) by analysis for specific *M. furfur* IgE (m70 and *Malassezia spp.* m227, ImmunoCap250, Thermo Fisher).

***Malassezia furfur* extracts:** *M. furfur* was grown in potato dextrose broth (Difco) containing Tween 40 (10 ml/L), olive oil (Merck, 2.5 ml/L) and glycerol (2.5 ml/L) at 32°C and 120 rpm. *M. furfur* cells were harvested by centrifugation at 3000 x *g* for 15 min. Cell pellets were resuspended in ice-cold PBS and transferred to lysis tubes (BioSpec Products) to disrupt cells at 1400 rpm for 40 sec with a FastPrep (MP Biomedicals). Extracts were centrifuged at 20.000 x *g* for 15 minutes to remove debris. Extracts were stored at -80 °C until use. For collecting secreted proteins, *M. furfur* was grown for three days as described above. This culture was used to inoculate a new culture (inoculum 1 % of the final culture volume). After three days of cultivation, cells were harvested by centrifugation and supernatants filtered (0.45-µm filter) to remove residual yeast cells. Secreted proteins were precipitated with 20% trichloroacetic acid (TCA) and 3 mg/ml dithiothreitol (DTT) overnight on ice. After centrifugation at 26.000 x g for 20 min, the precipitated proteins were dissolved in methanol and chloroform to further purify the proteins according to the Wessel-Flügge protocol for protein precipitation/purification [1]. Samples were air dried, dissolved and sonicated in PBS. Proteins were frozen at -80 °C until further use.

Basophil activation test: Basophils activation test was conducted based on a previous report [2]. Briefly, equal volumes of blood were incubated with serial dilutions of *M. furfur* extracts samples were stained with anti-CCR3-APC-Vio770 (Milentyi Biotec), anti-CD3-FITC (Biolegend), anti-CD63-APC (Biolegend) and anti-CD203c-PE (Beckman Coulter). Residual red blood cells were lysed (RBC lysis buffer (Biolegend)). Samples were centrifuged and pellets were resuspended in 0.5%BSA in PBS for flow cytometric analysis. Basophils were identified from lymphocyte populations as CCR3+ CD3- cells. Activations was assumed by the simultaneous expression of CD63 and CD203c.

Antigen-reactive T cell enrichment: Antigen-reactive T cell enrichment (ARTE) was performed as previously described [3-8]. Briefly, peripheral blood mononuclear cells (PBMCs) were freshly isolated from EDTA blood. 1-2x10e7 PBMCs were plated in RPMI-1640 medium (GIBCO), supplemented with 5% (v/v) human AB-serum (Sigma Aldrich, Schnelldorf, Germany) in 12-well cell culture plates and stimulated with 40ug/mL *Malassezia furfur* (DSM6170) for 7 h in presence of 1 µg/mL CD40 and 1 µg/mL CD28 pure antibody (both Miltenyi Biotec, Bergisch Gladbach, Germany). 1 µg/mL Brefeldin A (Sigma Aldrich) was added for the last 2 h. PBMC were labeled with CD154-Biotin followed by anti-Biotin MicroBeads (CD154 MicroBead Kit, Miltenyi Biotec) and magnetically enriched by two sequential MS columns (Miltenyi Biotec). Surface staining was performed on the first column, followed by fixation and intracellular staining on the second column. Frequencies of antigen-specific T cells were determined based on the cell count of CD154+ Tmem cells (CD4+ CD45RA- CD45RO+) after enrichment, normalized to the total number of CD4+ T cells applied on the column. For each stimulation, CD154+ background cells enriched from the non-stimulated control were subtracted (Fig S2). Cells were stained in different combinations of fluorochrome-conjugated antibodies. Viability 405/520 Fixable Dye (Miltenyi Biotec) was used to exclude dead cells. For intracellular staining cells were fixed and permeabilized with the Inside stain Kit (Miltenyi Biotec). Data were acquired on a LSR Fortessa (BD Bioscience, San Jose, CA, USA) or Northern Lights 3000 (Cytek Biosciences). FlowJo (Treestar, Ashland, OR, USA) software was used for analysis.

Western blot: For detection of immune-reactive *M. furfur* proteins, *M. furfur* extract was denatured and separated by 12% SDS-PAGE. *M. furfur* proteins were blotted onto nitrocellulose membranes. Membranes were blocked, washed and were incubated with patient serum (1:100). Membranes were

washed and incubated with anti-IgE-HRP (Novus Biologicals). Membranes were washed and immunoreactive bands were detected using the WesternBright Chemiluminescence substrate kit (Biozym scientific) according to the manufacturer's protocol. The established *Malassezia* allergens detected (www.allergen.org) are specified, including Mala f 2 and 3 (Figure 2).

***Malassezia spp.* quantification on the skin:** Skin swabs were taken on the neck and on the forearm directly distal to the crook of the elbow. DNA from swabs was isolated using the QIAamp UCP Pathogen Mini Kit according to manufacturer's instructions. DNA standards for *M. furfur* (DSM-6170), *M. sympodialis* (CBS-7222) *M. restricta* and *M. globosa* (both Westerdijk Fungal Biodiversity Institute) quantification were prepared by DNA isolation (DNeasy UltraClean Microbial Kit) from yeast cultures. Quantification of *Malassazia spp*. DNA was performed using TaqMan-based qPCR as previously described [9] using LightCycler® 480 Probes Master on a LightCycler® 480 System (Roche).

Enzyme-linked Immunosorbent Assay (ELISA)

M. furfur -specific Ig was detected through *M. furfur* extract coated 96-well plates (Nunc Maxisorb) after blocking with 2% BSA/PBS, incubation with serum (diluted in a range from 1:100 to 1:400) and immunoglobulin-specific detection using anti-IgA-HRP (Novus Biologicals) or alkaline phosphatase conjugated anti-IgG (Jackson ImmunoResearch). The colorimetric reaction was quantified photometrically. The O.D. were normalized to a standard curve of serially diluted pooled serum of two *Malassezia*-IgE-dominant patients. *Malassezia* crossreactivity IgE ELISA was determined using individual patient serum preincubated with the respective *Malassezia* extracts (2µg/ml) for 1 h followed by ELISA. 3% biotin-free BSA/PBS was used for blocking and detection of specific IgE was performed using biotin-conjugated clone HP6029 and streptavidin-AP. The colorimetric reaction was quantified photometrically.

Supporting References:

1. Wessel D, Flügge UI. A method for the quantitative recovery of protein in dilute solution in the presence of detergents and lipids. *Analytical Biochemistry.* 1984;138(1):141-143.
2. Behrends J, Schwager C, Hein M, Scholzen T, Kull S, Jappe U. Innovative robust basophil activation test using a novel gating strategy reliably diagnosing allergy with full automation. *Allergy.* 2021;76(12):3776-3788.
3. Bacher P, Heinrich F, Stervbo U, et al. Regulatory T Cell Specificity Directs Tolerance versus Allergy against Aeroantigens in Humans. *Cell.* 2016;167(4):1067-1078.e1016.
4. Bacher P, Hohnstein T, Beerbaum E, et al. Human Anti-fungal Th17 Immunity and Pathology Rely on Cross-Reactivity against Candida albicans. *Cell.* 2019;176(6):1340-1355.e1315.
5. Bacher P, Rosati E, Esser D, et al. Low-Avidity CD4(+) T Cell Responses to SARS-CoV-2 in Unexposed Individuals and Humans with Severe COVID-19. *Immunity.* 2020;53(6):1258-1271.e1255.
6. Bacher P, Schink C, Teutschbein J, et al. Antigen-Reactive T Cell Enrichment for Direct, High-Resolution Analysis of the Human Naive and Memory Th Cell Repertoire. *The Journal of Immunology.* 2013;190(8):3967-3976.
7. Saggau C, Martini GR, Rosati E, et al. The pre-exposure SARS-CoV-2-specific T cell repertoire determines the quality of the immune response to vaccination. *Immunity.* 2022;55(10):1924-1939.
8. Martini GR, Tikhonova E, Rosati E, et al. Selection of cross-reactive T cells by commensal and food-derived yeasts drives cytotoxic TH1 cell responses in Crohn's disease. *Nature medicine.* 2023;29(10):2602-2614.
9. Ilahi A, Hadrich I, Neji S, Trabelsi H, Makni F, Ayadi A. Real-Time PCR Identification of Six Malassezia Species. *Curr Microbiol.* 2017;74(6):671-677.

Supplementary information

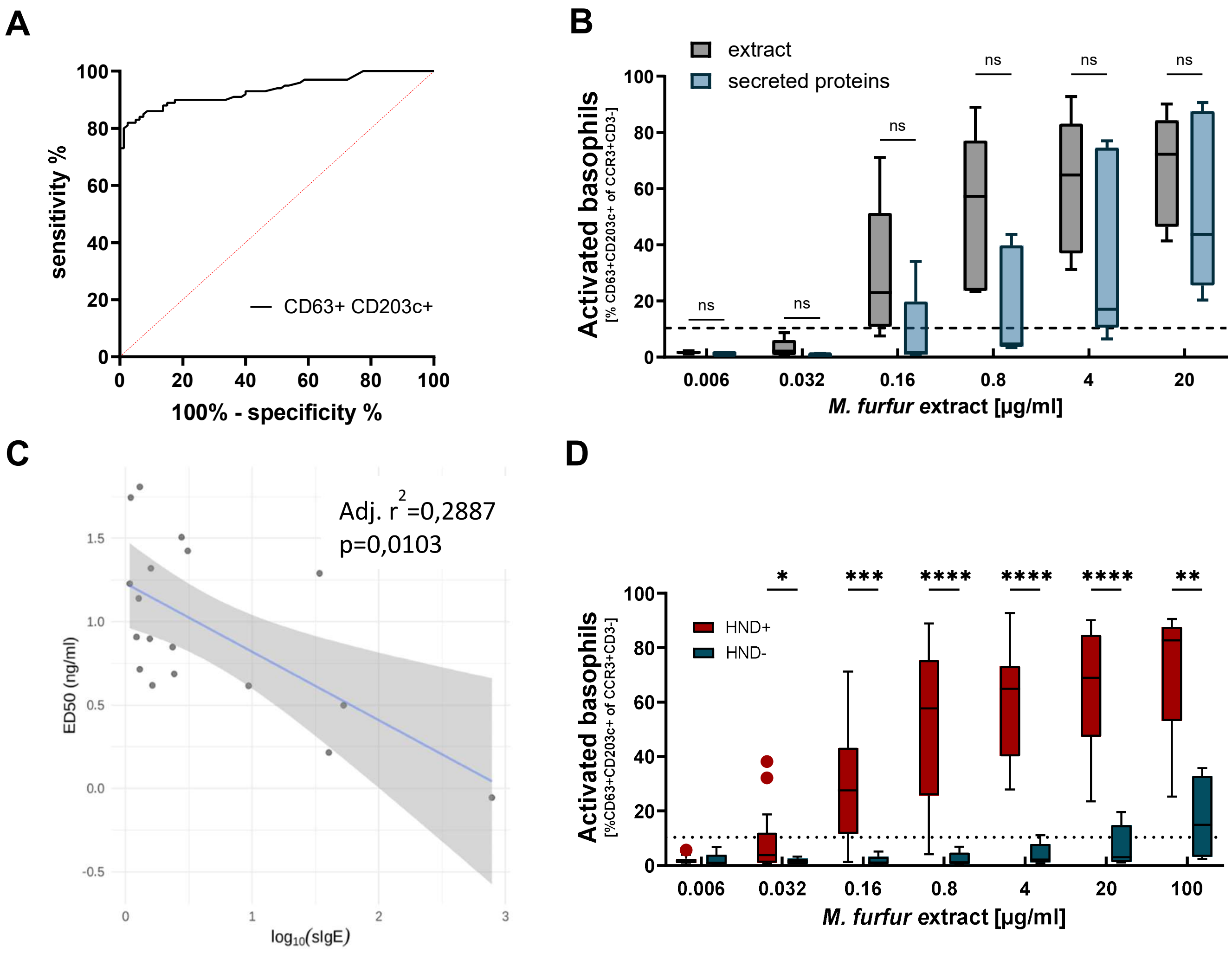

Figure S1: *Malassezia*-induced basophil activation test in HND. Peripheral full blood cells from HND and NA were incubated with titrated *M. furfur* extract and analysed after erythrolysis by flow cytometry regarding CD63 and CD203 expression on CCR3+CD3- basophils. (A) ROC curve analysis of CD63+CD203+ cells. (B) Frequency of activated basophils to titrated *M. furfur* extract and *M. furfur* soluble proteins in HND donors. (C) Correlation between EC50 of the basophils activation test and *M. furfur*-specific serum IgE levels (log_{10}) in HND patients. (D) Frequency of activated basophils from sensitized (HND+) and not sensitized (HND-) individuals with AD in response to titrated *M. furfur*-extract. Box plots represent the 25-75 percentils with range (5-95%). * = p<0,05; ** = p<0,01; *** = p<0,001; **** = p<0,0001.

Supplementary information

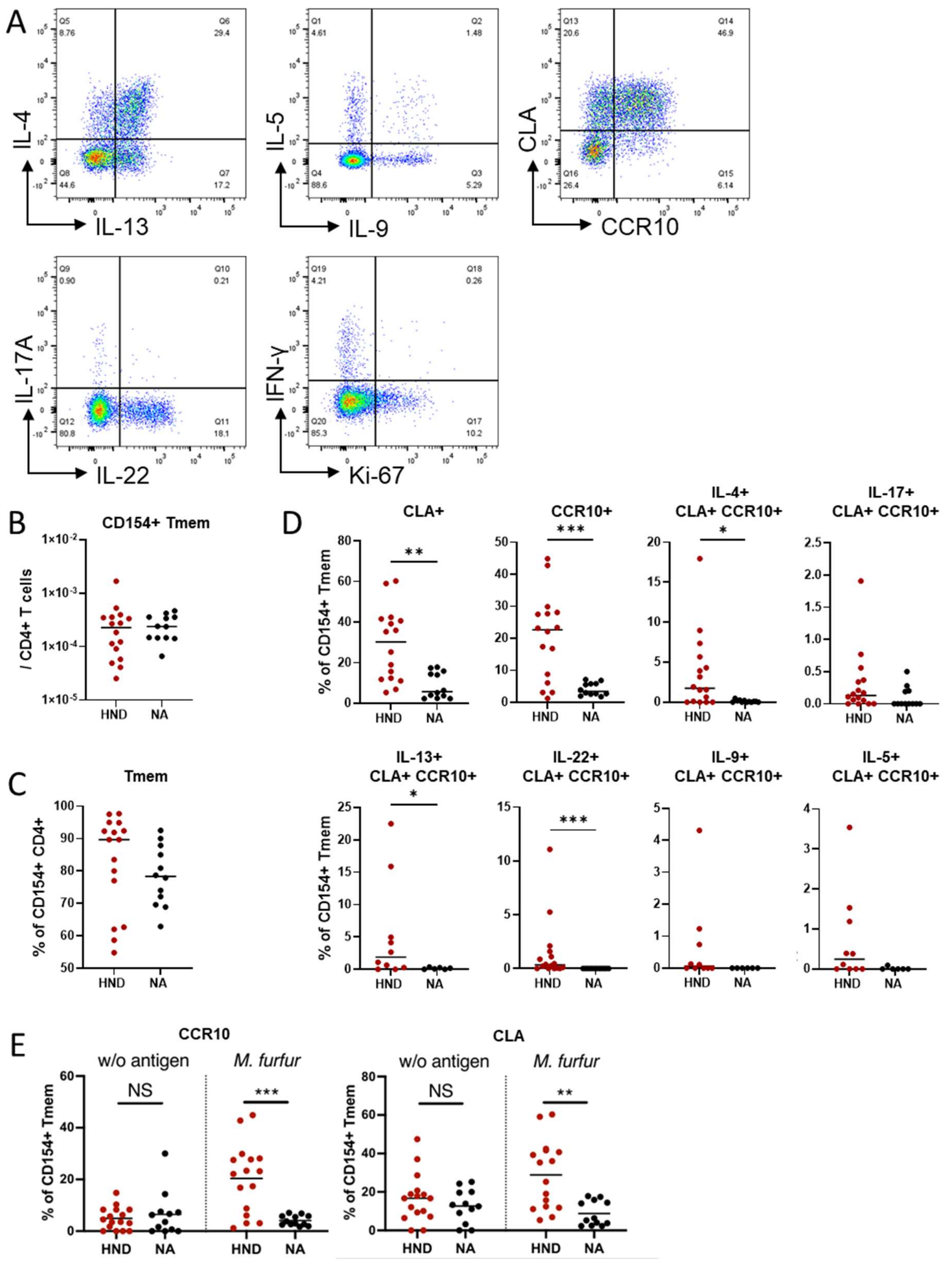

Figure S2: *M. furfur*-reactive memory CD4+ T cells in HND and NA individuals. *M. furfur*-Tmem cells were characterized by ARTE. The dot plots are gated on single CD4+ CD45RO+ CD45RA- CD154+ lymphocytes. (A) *Ex vivo* cytokine production. Graphs show one representative HND donor. (B) Absolute and (C) relative frequency of *M. furfur*-Tmem. (D) Statistical analysis of cytokine expression by skin homing of *M. furfur*-Tmem in HND donors and (E) *M. furfur*-Tmem cell frequencies expressing CCR10+ or CLA+ after stimulation compared to baseline (n=10-16) and NA (n=6-12). * = p< 0,05; ** = p< 0,01; *** = p<0,001

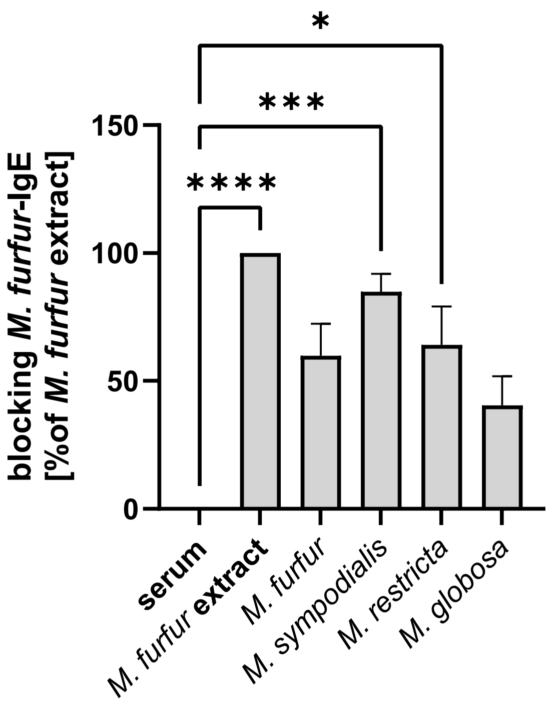

Figure S3: Blocking cross-reactivity between *Malassezia* spp. Inhibition assay of *M. furfur* IgE binding by ELISA. Serum was incubated with indicated yeast extracts or serum before added to the ELISA plate. n=10, 7 individuals. Results were normalized to sample with highest inhibition effect. Kruskal-Wallis with *<0,05; **<0,01; ***<0,001 and ****<0,0001.
